# Conditions Simulating Enlargement of the Liver.—III

**Published:** 1909-05-29

**Authors:** 


### Medicine.

### CONDITIONS SIMULATING ENLARGEMENT OF THE LIVER.—III.

### Pancreatic Tumours.

The points of distinction between a pancreatic and a hepatic enlargement are as follows:—

A pancreatic tumour is usually situated between the ensiform cartilage and the umbilicus. It does not as a rule occupy the right hypochondriac region unless it is a very large cyst—a so-called pancreatic cyst, which in many cases is really a cyst of the lesser omental sac—and in this case it may almost fill the whole abdominal cavity.

On account of its close proximity to the aorta a well-marked transmitted pulsation may be seen and felt in the tumour. Neither edge nor notch can be felt. A pancreatic tumour is deeply situated in the abdomen, is fixed, and does not move during respiration. A liver tumour is superficial, close under the abdominal parietes, and it moves freely with respiration. The stomach lies in front of a pancreatic tumour and behind an enlarged liver. If this point cannot be determined by the ordinary methods of examination, the stomach should be distended with gas by administering bicarbonate of potash and tartaric acid in separate solutions, after which its relations will nearly always become obvious. There is usually resonance over a pancreatic tumour unless it is very large, whilst a big liver is generally dull to light percussion. A large hydatid cyst of the left lobe of the liver is the tumour which is most likely to be mistaken for a pancreatic cyst.

### Omental Tumours.

The commonest omental conditions to simulate a big liver are those which result from inflammatory thickening, tuberculous peritonitis, and carcinoma. The mass may be more or less smooth with what feels like a well-defined hepatic edge, or it may be irregular, nodular, and large. The points of distinction are as follows:—

An omental tumour is apt to occupy the epigastric or umbilical regions. It may, however, lie across the abdomen, stretching from one hypochondrium to the other. It is superficial, and it moves slightly during respiration, unless it is adherent to the abdominal wall.

In the case of an omental tumour the observer's hand can usually be placed between it and the ribs, even though its upper limit cannot be determined. In the case of a liver enlargement the mass comes down close under the costal margin, and the hand cannot be inserted above it.

The stomach lies above an omental tumour, but behind a liver tumour, so that in the former case there is resonance above the tumour between it and the ribs, whilst in the case of an enlargement of the liver there is dulness which is continuous with the normal hepatic dulness. Tuberculous disease of the omentum usually occurs as a part of tuberculous peritonitis, of which there may be separate evidence, such as redness, œdema, and purulent discharge in the neighbourhood of the umbilicus, and so on. Calmette's reaction may be tried and found to be positive. There may be ascites requiring the performance of paracentesis abdominis, and tubercle bacilli may be found in the fluid, either by direct examination or by animal inoculation.

*Fæcal Accumulation in the Hepatic Flexure or in the Transverse Colon* may be mistaken for an enlarged liver. To distinguish the two the following points should be attended to:—

In fæcal accumulation cases there is usually a history of obstinate constipation and possibly of attacks simulating intestinal obstruction.

The tumour is of an irregular form, generally more or less cylindrical, the shape varying with the amount of fæces present.

No definite edge or notch is to be made out as a rule, unlike the case of the liver.

The mass may sometimes feel almost like putty, the fingers being able to mould it into various shapes.

The hand is nearly always able to get between the mass and the costal margin. This is hardly ever the case with an enlarged liver.

On light percussion the fæcal mass may give a dull note, but percussion of any force will nearly always give a more resonant note with impacted fæces than it would with a liver enlargement.

*Tumours of the Abdominal Wall or Rigidity of the Recti Muscles* may also be mistaken for enlarged liver. In the case of a tumour in the abdominal wall the relation of the mass to the abdominal muscles must be determined, and this is best accomplished by getting the patient to lie on his back and then to raise his neck and head from the pillow; the abdominal muscles will at once become firmly contracted, and it can then be ascertained whether the tumour has become more or less prominent than before. The test is not absolutely certain, of course, for the mass may be in the abdominal wall and yet behind the muscles, in which case it would become less obvious under the above procedure precisely as an intra-abdominal mass would. If it becomes more prominent, however, it is in the abdominal wall.

A tumour of the wall does not move up and down during respiration, whereas a tumour of the liver moves freely downwards when the patient inspires deeply, unless indeed the hepatic mass has become fixed to the abdominal wall.

The patient should be examined in the knee-elbow position. A growth in the abdominal wall becomes more prominent, and it may be felt to have no deep connections, whereas an intra-abdominal tumour, unless it is adherent to the abdominal wall, will become less definite.

In many cases it is quite impossible to arrive at a definite conclusion unless an examination is made under a general anæsthetic. In the case of rigidity of the muscles the latter become relaxed under the influence of the anæsthetic, and a much more thorough investigation of the abdomen by palpation can be made.

